# Evaluation of multiple immune cells and patient outcomes in esophageal squamous cell carcinoma

**DOI:** 10.3389/fimmu.2023.1091098

**Published:** 2023-02-20

**Authors:** Hui Wang, Chanjuan Su, Ziteng Li, Changchun Ma, Liangli Hong, Zhe Li, Xiaonan Ma, Yien Xu, Xiaolong Wei, Yiqun Geng, Weifeng Zhang, Penghao Li, Jiang Gu

**Affiliations:** ^1^ Provincial Key Laboratory of Molecular Pathology and Personalized Medicine, Center of Collaborative and Creative Center, Department of Pathology and Pathophysiology, Shantou University Medical College, Shantou, Guangdong, China; ^2^ Department of Radiation Oncology, Affiliated Cancer Hospital, Shantou University Medical College, Shantou, Guangdong, China; ^3^ Department of Pathology, Affiliated Cancer Hospital, Shantou University Medical College, Shantou, Guangdong, China; ^4^ Jinxin Research Institute for Reproductive Medicine and Genetics, Xinan Hospital for Maternal and Child Health Care, Chengdu, China

**Keywords:** immunoglobulin G4, CD8^+^ T lymphocyte, CD4^+^ T lymphocyte, CD4^+^Foxp3^+^ regulatory T lymphocyte, esophageal squamous cell carcinoma, prognosis

## Abstract

Recent reports indicate that immune cells in solid cancers have significant predictive and therapeutic value. IgG4 is a subclass of IgG and we recently found that it exerted an inhibitory effect in tumor immunity. We aimed to assess the significance of IgG4 and T cell subtypes in tumor prognosis. We investigated the density, distribution and relationship of five immune markers CD4, CD8, Foxp3, IL-10 and IgG4 with multiple immunostaining method in 118 esophageal squamous cell carcinoma (ESCC) together with clinical data. The relationship among different immune cell types and with clinical data were analyzed with Kaplan-Meier survival analysis and Cox proportional hazards model to identify independent risk factors among immune and clinicopathological parameters. Five-year survival rate of these patients treated with surgery reached 61%. Higher number of CD4^+^ plus CD8^+^ T cells predicted better prognosis (p=0.01) in tertiary lymphoid structure (TLS) and could add to the value of TNM staging. Density of the newly identified immune inhibitor IgG4^+^ B lymphocytes was found positively correlated to that of CD4^+^ cells (p=0.02) and IL-10^+^ cells (p=0.0005), but number of infiltrating IgG4^+^ cells by itself was not an independent factor for prognosis. However, increased serum concentration of IgG4 indicated a poor prognosis of ESCC (p=0.03). 5-year survival rate of esophageal cancer after surgery has been significantly improved. Increased T cells in TLS predicted better survival, suggesting that T cells in TLS may actively participate in anti-tumor immunity. Serum IgG4 could be a useful predictor of prognosis.

## Introduction

Esophageal cancer is a malignant tumor with difficulty in early diagnosis. In 2020, there were 604100 new cases of esophageal cancer and 544076 deaths globally. In contrast to the predominant adenocarcinoma in western countries, more than 90% of esophageal cancer in most Asian countries was classified as squamous cell carcinoma (1–3). The 5-year survival rate has raised to nearly 40% in China in recent years due to improvement in early diagnosis and treatment but still has a lot to desire (4). Conducting detailed molecular and immunopathology investigations is necessary to explore new strategies for managing esophageal cancer.

Tumor pathological TNM (pTNM) staging is widely used to evaluate disease progression and cancer stage (5–7). There is great heterogeneity in the phenotype, function, and density of immune cells in tumor microenvironment (TME) (8–10). In recent years, immune score has been found useful to predict tumor survival and prognosis (11). Studying the type, density, distribution, and functional phenotype of immune cells infiltrated in TME is a good supplement to pTNM staging. In colorectal cancer, in particular, studies have shown that recurrence time and overall survival are closely related to immune cell density on the invasive edge of tumor masses (12–14).

T cell-mediated adaptive immunity, including CD8 cytotoxic T cell, has attracted extensive attention, and relevant immunotherapeutic drugs have been developed, such as monoclonal antibodies against PD-1 and CTLA-4 (15–18). Immunosuppressive cells, such as Foxp3 positive T regulatory cells, myeloid-derived suppressor cells, tumor associated macrophages, and cancer-associated fibroblasts are generally associated with negative prognostic values (19).

IgG4 is the least abundant subclass accounting for 5% of the four IgG subclasses in human serum. It has the weakest ability among the four subclasses to bind the Fc receptor of effector immune cells and activate complements. This leads to its low ability to activate complement and macrophage-mediated antibody-dependent cellular cytotoxicity (20–22). In tumor immunity, IgG4 is an immunosuppressive molecule (23) and present in a subpopulation of B lymphocytes. In recent studies, IgG4 was reported to increase in serval tumor types including gastric cancer (24), melanoma (25), esophageal cancer (26) and extrahepatic cholangiocarcinoma (27). In the presence of IL-10, IgG4 could promote immunosuppression and inhibit inflammatory reactions (28, 29).

Up to now, there has been no study comparatively evaluating immunosuppressive and immunopromoting cells, and compare them with other clinicopathological parameters. In this study, we analyzed five immune markers including CD8, CD4, Foxp3, IgG4, and IL-10, together with other clinical predictive indicators. We aimed to find a more accurate and balanced assessment of multiple immune cell types in TME to supplement the TNM scoring system that has been used for many years in pathological diagnosis.

## Materials and methods

### Study population

A cohort of 118 patients diagnosed as ESCC and received resection surgery and postoperative therapies at the Cancer Hospital of Shantou University Medical College between October 2013 and March 2017, were included in our study. The inclusion criteria were as follows: 1) histological confirmed primary ESCC patients and the tumor stages were updated according to the American Joint Committee on Cancer Staging Manual (8th edition); 2) no preoperative radiotherapy or chemotherapy; 3) no other malignant diseases; 4) no autoimmune deficiency or immune-related diseases; 5) available block of formalin-fixed and paraffin-embedded (FFPE) tumor tissue; 6) available and comprehensive clinical information and clinical follow-up data including survival Status and survival time. Two interested endpoints were designated for the study. Overall survival (OS) refers to the time from surgery to death due to any cause, and progression-free survival (PFS) refers to the time from surgery to the first observation of disease progression or death due to any cause. The serum markers of 82 cases were originally collected from the SUMC affiliated tumor hospital in 2016-2017, of which 72 cases obtained follow-up information, and relevant clinical analysis was carried out. The concentrations and detection methods of their sera IgG4 were described in our previous publication (26).

### Tissues and staining process

FFPE tissue blocks were cut into 4μm serials slides for hematoxylin & eosin (H&E) staining, immunohistochemistry (IHC) staining and multi-color Immunofluorescence (mIF). For detailed IHC experimental procedures, please refer to our previous publication (30).

Multi-color immunofluorescence staining and scoring

Five markers CD4, CD8, FOXP3, IL-10 and IgG4 were stained on tissue from surgical samples of 118 ESCC specimens employing TSA-RM-24259 (50T) kits from PANOVUE according to the manufacturer’s instructions. The dilution and fluorescence of antibodies were shown in **Supplementary Table 1**. The protocols were described briefly below. First, slides were deparaffinized and rehydrated as for conventional IHC. Then, 4% paraformaldehyde was used for tissue re-fixation for 10 min at room temperature followed by antigen retrieval the same as for IHC. After cooled down and washed with 1X TBST 3 times 5min each, slides were further incubated with 10% horse serum for 10 min and then primary antibody for 30min at room temperature. Next, slides were performed with a second antibody for 10 min and PPD fluorescent dye solution, diluted at 1:100 with signal amplifying fluid of the kit, for 10 min. Then, the sections went through antigen retrieval again and entered into next cycle until the 5 markers signals were stained. Finally, the slides were incubated with DAPI and covered with coverslips, and the slides were then ready for multispectral microscopic imaging and automatic cell counting.

**Figure 1:**
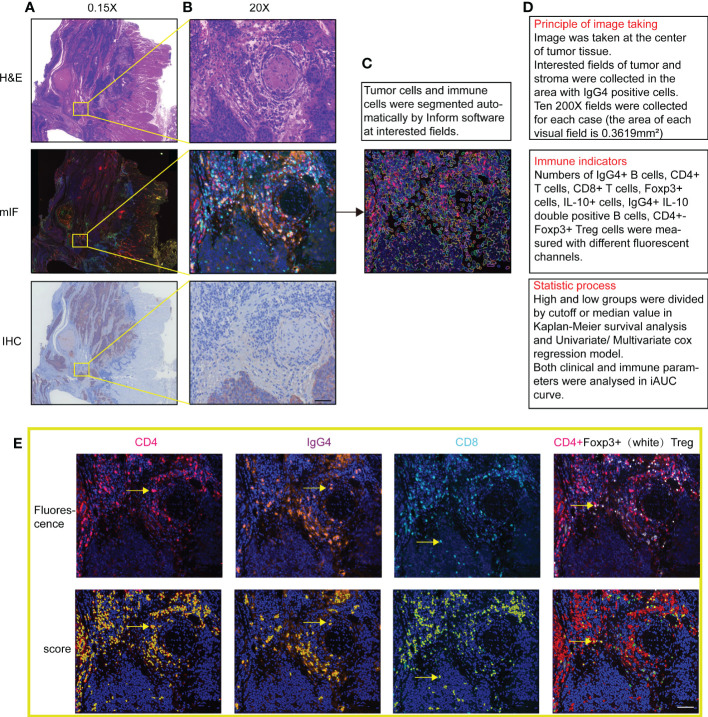
**(A, B)** Images of multi-color immunofluorescence staining with H&E staining and immunohistochemistry in 1.5X and 200X magnification in image scanning software. **(C)** Cell nuclear and membrane automatically recognized with software in fluorescence staining fields. **(D)** Three steps of drawing, counting and statistics with the software. **(E)** Shows both actual fluorescent staining and automatic score of positive signals of the same fields. Positive counting shows in yellow by Inform software. Scale bar=60μm.

Multispectral microscopic imaging employed PerkinElmer Vectra device and automatic counting used inForm^®^ V2.2 advanced image analysis system. More than 5 random high resolutions (200X) microscope fields of tumor parenchyma or TLS were acquired for scoring, which contained tumor and stroma areas or with IgG4 positive cells. Based on computer intelligent recognition algorithm, the software distinguished differently defined areas and identified every single cell according to the DAPI signal of cell nucleus. In the end output, the software displays single positive rates of 5 markers individually and double positive rates of CD4 plus Foxp3. The process of tissue staining and positive cell counting are shown in **Figure 1**.

### Statistical analyses

The data obtained were analyzed statistically. All results were analyzed with IBM SPSS statistical version 26.0, GraphPad Prism version 9.0.0, R project version 4.1.2, and RStudio version 2021.09.1 + 372. Pearson correlation coefficients were used to test the correlation of different markers in the same area. After the Homogeneity test, Brown-Forsythe test and Levin variance equivalence test were performed to distinguish the variance of markers in different groups, according to the T, N, G, pTNM stages and family history, followed with Tukey’s multiple comparisons test for differences between groups. When the homogeneity of variance is not met, Kruskal-Wallis test was conducted for non-parametric test. In advance of survival analysis, all experimentally observed parameters, which were continuous variables, were divided into two groups, low and high groups, depending on the optimal cut-off estimated from survival ROC curves (“survival ROC”, R package). Kaplan-Meier curves and log-rank test were conducted to compare the survival rates in separate low and high groups. Cox proportional hazards models were conducted to distinguish the correlation between the experimental indicators and the survival times prior to Schoenfeld Individual test (“survival”, R package). Time-dependent ROC curve and the concordance index (C-index) demonstrated the predictive efficacies for different models (“time ROC”, R package). All statistical tests were two-sided. The condition of a P-value < 0.05 was set as statistically significant.

## Results

### Clinicopathological characteristics of esophageal cancer patients

The clinical characteristics of the patients enrolled in the study are presented in **Table 1**. One hundred eighteen (118) patients with ESCC were eligible for inclusion, of whom 82.2% (97/118) were male and 17.8% (21/118) were female, with an average age of onset of 59.86 years. Among the enrolled patients, 71.2% had a history of tobacco use and 45.8% had a history of alcohol use. Among them, the incidence of tumors in the middle esophagus was the highest, reaching 61.9%. All 118 patients underwent esophagectomy, of which 8% were TNM stage I and 40.7% were stage III. 16.9% of all patients had a family history, 29.7% received postoperative radiotherapy, and 34.7% received postoperative chemotherapy. None of the enrolled patients received preoperative chemoradiotherapy or immunotherapy. The 5-year survival rate of 118 cases of ESCC patients who received surgery resection was 61%, and that of 72 esophageal cancer patients with surgical resection or radiochemotherapy was 43%.

**Table 1:** Clinicopathological characteristics.

Clinical Characteristics	Cohort (n=118)
Gender	
Male	97 (82.2%)
Female	21 (17.8%)
Age at surgery (years)	
N	118 (100%)
Mean	59.86
Range	42-77
Tabacco use	
Yes	84 (71.2%)
No	34 (28.8%)
Alcohol use	
Yes	54 (45.8%)
No	64 (54.2%)
Tumor location	
L	16 (13.6%)
M	73 (61.9%)
U	27 (22.9%)
Multiple	1 (0.8%)
Esophagogastric junction	1 (0.8%)
T stage	
T1	9 (7.6%)
T2	11 (9.3%)
T3	48 (40.7%)
T4	50 (42.4%)
N stage	
N0	51 (43.2%)
N1	36 (30.5%)
N2	23 (19.5%)
N3	8 (6.8%)
M stage	
M0	118 (100%)
M1	0 (0.0%)
Histologic Grade	
G1	53 (44.9%)
G2	57 (48.3%)
G3	8 (6.8%)
pTNM stage	
I	8 (6.8%)
II	35 (29.7%)
III	48 (40.7%)
IV	27 (22.9%)
Genetic disorder	
Yes	20 (16.9%)
No	98 (83.1%)
Postoperative radiotherapy	
Yes	35 (29.7%)
No	83 (70.3%)
Postoperative chemotherapy	
Yes	41 (34.7%)
No	77 (65.3%)
Survival status	
0	71 (60.2%)
1	47 (39.8%)

### Density and distribution of infiltrating immune cells

Most of the immune cells were distributed in the tumor stroma, and only a small group of CD4/CD8 T cells were in the tumor parenchyma. Most of the specimens showed CD8^+^ and CD4^+^ T cell infiltration. 77% (91/118) cases had high number CD4^+^ T cell infiltration (cutoff=345 cells/20X HPF). 15% (18/118) cases had high number CD8^+^ T cell infiltration (cutoff=394 cells/20X HPF). 79% (94/118) cases had high number FoxP3^+^ T cell infiltration (cutoff=13 cells/20X HPF). 60% (71/118) cases had high number IgG4^+^ plasma cell infiltration (cutoff=10 cells/20X HPF).

### Correlation and variations of the five immunological parameters in tumor

**Figure 2:**
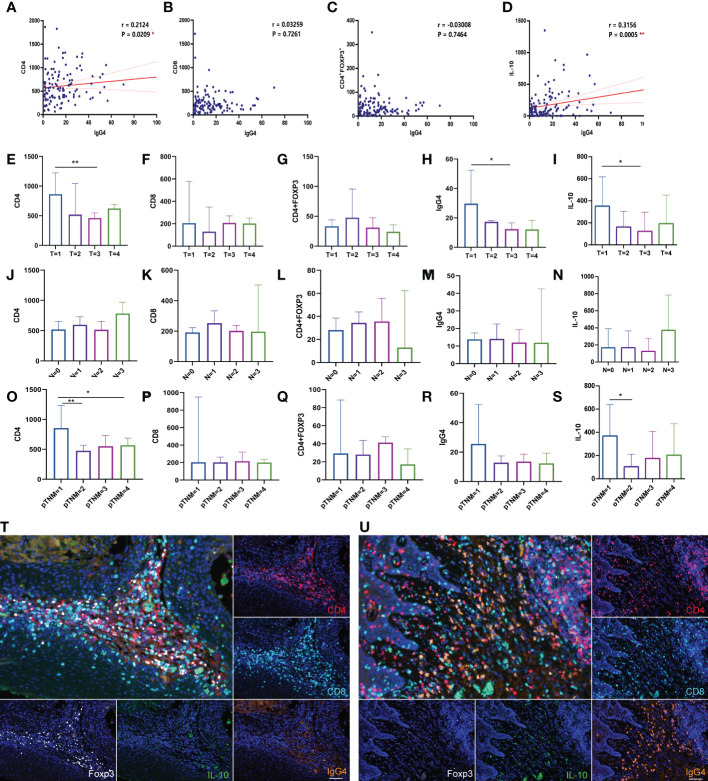
**(A–D)** Correlation analysis of IgG4, with CD4, CD8, Foxp3 and IL-10 positive cells (n=118). **(E–S)** Comparison of CD4, CD8, Treg, IgG4 and IL-10 in different T stages, N stages, pTNM stages (n=118). **(T, U)** The two figures show the typical multicolor immunofluorescence staining image located at high Foxp3 or IgG4 positive cells expressing area. Scale bar=60 μm. *p<0.05, **p<0.01.

T cell-mediated antitumor immunity has important implications for patient survival. CD4 and CD8 cells are the main T cell subsets. CD8^+^ T lymphocytes mainly play a cytotoxic function (31). CD4^+^ FoxP3^+^ T regulatory cells (Tregs) are T helper cells play important regulatory and immunosuppressive functions (32). Based on staining results, we analyzed the correlation between IgG4-positive cells and other cell types and found that IgG4 with CD4/IL-10 positive cells were positively correlated in number with statistical significance (p= 0.02, p=0.0005). IgG4^+^ was positively or negatively correlated with CD8^+^ or Treg cell density, but the relationship was not statistically significant (**Figures 2A–D**). The distribution of CD4^+^, CD8^+^,IgG4^+^, IL-10^+^ cells in different T stage, N stage and pTNM stage had their own distribution characteristics, but the difference was not statistically significant, as shown in **Figures 2E–S**. However, in two by two comparisons, CD4^+^cells in some Tumor stages (T1 vs T3, **, p=0.006) and pTNM stages (I vs II **, p=0.004, I vs IV, * p=0.04) were statistically significant. Also IgG4^+^ cells in some T stages (T1 vs T3, *, p=0.03) was statistically significant. IL-10^+^ cell numbers were higher in T1 stage than in T3 stages (T1 vs T3, *, p=0.02) and also higher in I than II (I vs II, *, p=0.04). **Figures 2T, U** show the staining results of typical fields of multiple fluorescence staining. Different from high density of CD4^+^ and CD8^+^ cells in tumor TME, the infiltrating number of FoxP3 or IgG4 positive cells was low, and the expression pattern variated in different cases.

### K-M survival analysis of clinicopathological and immunoparameters

**Figure 3:**
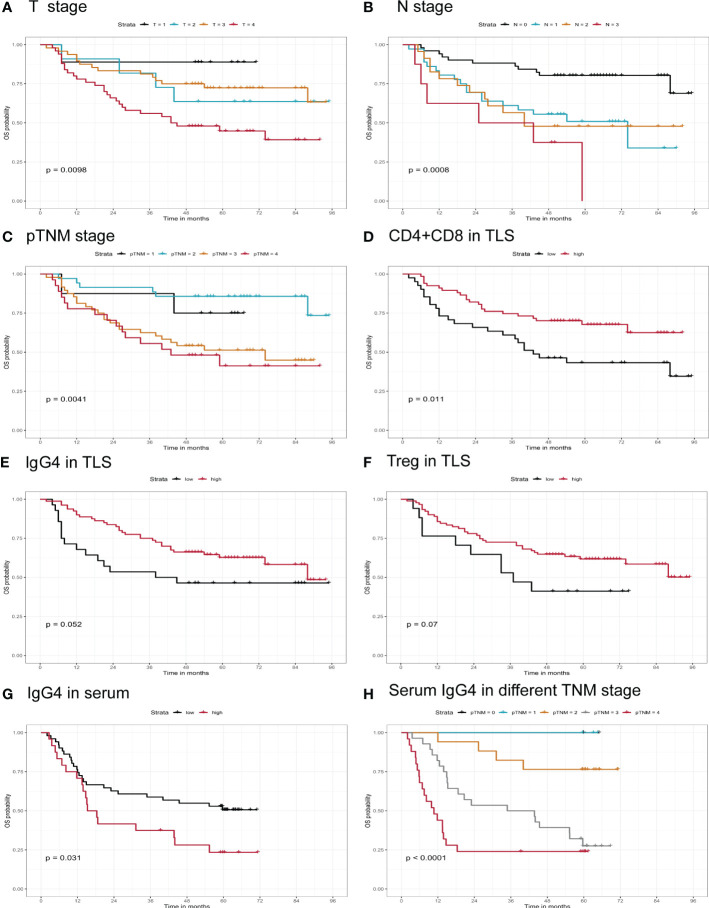
Correlation of infiltrating immune markers and patients’ clinical survival time. **(A–F)** Overall survival of patients grouped by different T stage, N stage, pTNM stage, CD4+CD8, IgG4, Treg in TLS infiltrating status (n=118). **(G, H)** Serum IgG4, serum IgG4 in different TNM stage in Kaplan-Meier survival analysis (n=72).

Survival prognostic analysis was performed on the clinical and pathological indicators of the patients, and it was found that in T (p=0.009), N (p=0.0008), and pTNM stages (p=0.004), the lower the stage, the longer the survival time of the patient (shown in **Figures 3A–C**). We performed K-M analysis in tumor parenchyma and TLS. We observed that the higher the CD4 density, the better the prognosis. The higher the infiltration density of CD4 + CD8 in TLS (p=0.01, cutoff= 1074.943733 cells/200xHPF), the longer the survival time of patients, suggesting that the total density of T cells plays a key role in tumor TLS (shown in **Figure 3D**). Similarly, the higher the density of IgG4^+^ or Treg cells infiltrating the tumor TLS, the longer the patient’s survival time, but without reaching statistical significance (p=0.05; p=0.07) (shown in **Figures 3E, F**). This indicates that the increased cytotoxic T and Treg cells, and increased IgG4 positive B cells have a positive effect on the anti-tumor immunity. In addition, we followed up 82 cases of esophageal cancer randomly collected from 2016 to 2017, of which 6 cases were lost to follow-up, and analyzed the survival prognosis of 72 cases with the concentration of serum IgG4. It was found that the higher the serum IgG4, the worse the prognosis (p=0.03, Cutoff=12% IgG4/IgG). When analyzed the pathological stage of the same group of patients, it was found that the higher the stage, the shorter the survival time, and there was a marked statistical significance (p<0.0001) (shown in **Figures 3G, H**).

In addition, the influence of the patient’s family history, personal history treatment process, and IL-10 cell infiltration on the prognosis of ESCC was analyzed (shown in **Supplementary Figure 1**). It was found that there was no significant difference in survival time between patients who received postoperative chemotherapy(p=0.46) or radiotherapy(p=0.06) and patients who did not (shown in **Supplementary Figures 1C, D**). Alcohol consumption played a certain role in the prognosis of ESCC patients. The more alcohol used, the shorter the survival time of patients (shown in **Supplementary Figure 1E**).

### Analysis of independent risk factors for ESCC survival

In order to test whether the clinicopathological and immune indicators are independent risk factors in this cohort, univariate and multivariate cox regression analysis were performed on all clinical parameters and immune factors. We found that in univariate cox regression analysis, alcohol consumption is a risk factor for esophageal cancer (OS, HR=2.01, p=0.019). And the higher the stage of T stage (T4 vs T1, PFS, HR=8.5, p=0.035), N stage (N3 vs N0, OS, HR=5.8, p=0.001), and pTNM stage (IV vs I, PFS, HR=4.7, p=0.037), the higher the risk of poor prognosis. In addition, postoperative radiotherapy (PFS, HR=2.1, p=0.006) or chemotherapy (PFS, HR=2.42, p=0.001) also increased the risk of poor prognosis. Increased CD4 (OS, HR =0.395, p=0.007) and CD4+CD8 (OS, HR=0.478, p=0.013) in tumor TLS were protecting factor for survival, but with limited clinical significance (shown in **Table 2**).

**Table 2:** Univariate Cox regression analysis.

Variable	OS			PFS		
	HR (95% CI)	p-Value*	C-Index (95% CI)	HR (95% CI)	p-Value*	C-Index (95% CI)
Gender			0.526 (0.466-0.586)			0.523 (0.469-0.576)
Male	1.0 (reference)			1.0 (reference)		
Female	0.546 (0.216-1.380)	0.201		0.641 (0.290-1.416)	0.272	
Age			0.553 (0.479-0.626)			0.527 (0.459-0.595)
≤60	1.0 (reference)			1.0 (reference)		
>60	1.583 (0.888-2.825)	0.120		1.302 (0.770-2.201)	0.326	
Tabacco use			0.525 (0.456-0.595)			0.510 (0.447-0.574)
No	1.0 (reference)			1.0 (reference)		
Yes	1.489 (0.740-2.996)	0.264		1.207 (0.659-2.209)	0.543	
Alcohol use			0.579 (0.506-0.652)			0.548 (0.480-0.616)
No	1.0 (reference)			1.0 (reference)		
Yes	2.012 (1.123-3.606)	0.019*		1.524 (0.901-2.578)	0.116	
T stage			0.628 (0.552-0.704)			0.636 (0.567-0.704)
T2 vs T1	3.142 (0.350-28.195)	0.306		3.445 (0.385-30.848)	0.269	
T3 vs T1	2.539 (0.333-19.360)	0.369		3.587 (0.478-26.902)	0.214	
T4 vs T1	6.262 (0.851-46.079)	0.072		8.548 (1.168-62.572)	0.035*	
N stage			0.647 (0.571-0.722)			0.694 (0.628-0.761)
N1 vs N0	3.037 (1.428-6.457)	0.004*		3.142 (1.531-6.448)	0.002*	
N2 vs N0	3.033 (1.336-6.885)	0.008*		4.285 (2.018-6.448)	0.000*	
N3 vs N0	5.865 (2.153-15.976)	0.001*		12.364 (4.935-30.977)	0.000*	
G stage			0.514 (0.438-0.590)			0.530 (0.460-0.600)
G2 vs G1	0.973 (0.535-1.771)	0.929		1.019 (0.585-1.775)	0.948	
G3 vs G1	1.445 (0.494-4.226)	0.501		2.112 (0.860-5.189)	0.103	
pTNM			0.645 (0.570-0.719)			0.678 (0.611-0.744)
II vs I	0.601 (0.121-2.992)	0.534		0.757 (0.157-3.652)	0.729	
III vs I	2.281 (0.538-9.672)	0.263		2.815 (0.668-11.850)	0.158	
IV vs I	2.746 (0.627-12.03)	0.180		4.707 (1.097-20.200)	0.037*	
Postoperative radiotherapy			0.555 (0.486-0.624)			0.583 (0.520-0.647)
No	1.0 (reference)			1.0 (reference)		
Yes	1.750 (0.976-3.138)	0.060		2.101 (1.231-3.585)	0.006*	
Postoperative chemotherapy			0.504 (0.435-0.574)			0.599 (0.534-0.663)
No	1.0 (reference)			1.0 (reference)		
Yes	1.256 (0.701-2.252)	0.443		2.424 (1.431-4.103)	0.001*	
Family history			0.507 (0.450-0.564)			0.509 (0.457-0.561)
No	1.0 (reference)			1.0 (reference)		
Yes	1.027 (0.480-2.197)	0.946		1.094 (0.552-2.169)	0.796	
CD4			0.571 (0.505-0.638)			0.551 (0.491-0.611)
low	1.0 (reference)			1.0 (reference)		
high	0.485 (0.265-0.889)	0.019*		0.579 (0.327-1.024)	0.060	
CD8			0.551 (0.503-0.599)			0.534 (0.490-0.578)
low	1.0 (reference)			1.0 (reference)		
high	0.328 (0.102-1.055)	0.061		0.580 (0.248-1.352)	0.207	
CD4+CD8			0.555 (0.484-0.626)			0.527 (0.462-0.593)
low	1.0 (reference)			1.0 (reference)		
high	0.605 (0.323-1.132)	0.116		0.802 (0.464-1.386)	0.429	
Treg			0.559 (0.495-0.623)			0.551 (0.494-0.607)
low	1.0 (reference)			1.0 (reference)		
high	0.528 (0.277-1.007)	0.052		0.564 (0.311-1.023)	0.059	
IgG4			0.560 (0.486-0.634)			0.535 (0.467-0.604)
low	1.0 (reference)			1.0 (reference)		
high	0.729 (0.408-1.302)	0.285		0.849 (0.498-1.450)	0.550	
IL-10						
low	1.0 (reference)		0.555 (0.504-0.606)			0.516 (0.462-0.570)
high	0.575 (0.257-1.287)	0.178		0.868 (0.448-1.681)	0.674	
CD4 in TLS			0.562 (0.503-0.622)			0.538 (0.486-0.590)
low	1.0 (reference)			1.0 (reference)		
high	0.395 (0.201-0.779)	0.007*		0.535 (0.276-1.037)	0.064*	
CD8 in TLS			0.529 (0.482-0.576)			0.520 (0.478-0.562)
low	1.0 (reference)			1.0 (reference)		
high	0.460 (0.143-1.482)	0.193		0.663 (0.264-1.663)	0.381	
CD4+CD8 in TLS			0.595 (0.522-0.668)			0.559 (0.492-0.627)
low	1.0 (reference)			1.0 (reference)		
high	0.478 (0.267-0.856)	0.013*		0.602 (0.354-1.023)	0.061*	
Treg in TLS			0.550 (0.490-0.609)			0.553 (0.499-0.606)
low	1.0 (reference)			1.0 (reference)		
high	0.526 (0.260-1.064)	0.074		0.498 (0.262-0.947)	0.033*	
IgG4 in TLS			0.563 (0.489-0.637)			0.535 (0.465-0.604)
low	1.0 (reference)			1.0 (reference)		
high	0.722 (0.404-1.291)	0.272		0.876 (0.515-1.490)	0.625	
						

*Wald p Value.

Subsequently, in order to verify whether the included indicators are independent risk factors affecting the survival of esophageal cancer, a combined multivariate survival analysis was conducted. It was found that among all clinical indicators, only N stage (PFS, HR=5.2, p=0.004) is still a dangerous risk factor for the survival of tumor patients, while immune indicators, such as CD4 in TLS (OS, HR=0.317, P=0.008) was independent protective factors, and its value in clinical application needs further investigation (shown in **Table 3**).

**Table 3:** Multivariate Cox regression analysis.

Variable	OS			PFS		
	HR (95% CI)	p-Value *	C-Index	HR (95% CI)	p-Value *	C-Index
**Multivariable stratified Cox model of clinical parameters**			0.713			0.726
T stage						
T2 vs T1	3.114 (0.334-29.057)	0.319		2.829 (0.306-26.194)	0.360	
T3 vs T1	2.318 (0.297-18.070)	0.422		3.220 (0.419-24.750)	0.261	
T4 vs T1	4.246 (0.532-33.882)	0.172		4.255 (0.543-33.334)	0.168	
N stage						
N1 vs N0	2.896 (1.320-6.357)	0.008*		2.537 (1.208-5.328)	0.014*	
N2 vs N0	3.641 (1.316-10.075)	0.013*		3.267 (1.292-8.260)	0.012*	
N3 vs N0	2.971 (0.871-10.135)	0.082		5.226 (1.700-16.061)	0.004*	
Alcohol use						
No	1.0 (reference)			1.0 (reference)		
Yes	1.652 (0.886-3.080)	0.114		0.932 (0.525-1.655)	0.809	
Postoperative radiotherapy						
No	1.0 (reference)			1.0 (reference)		
Yes	1.635 (0.833-3.208)	0.153		1.928 (1.030-3.607)	0.040*	
Postoperative chemotherapy						
No	1.0 (reference)			1.0 (reference)		
Yes	0.447 (0.218-0.918)	0.028*		1.190 (0.626-2.262)	0.595	
**Multivariable stratified Cox model of clinical parameters with immunomicroenviroment markers**			0.743			0.732
T stage						
T2 vs T1	2.008 (0.200-20.183)	0.554		2.103 (0.215-20.550)	0.523	
T3 vs T1	1.271 (0.155-10.423)	0.823		2.282 (0.290-17.958)	0.433	
T4 vs T1	2.685 (0.326-22.086)	0.358		2.940 (0.365-23.658)	0.311	
N stage						
N1 vs N0	4.158 (1.811-9.548)	0.001*		3.279 (1.505-7.143)	0.003*	
N2 vs N0	5.242 (1.808-15.196)	0.002*		3.798 (1.425-10.128)	0.008*	
N3 vs N0	6.237 (1.738-22.387)	0.005*		9.605 (2.937-31.409)	0.000*	
Alcohol use						
No	1.0 (reference)			1.0 (reference)		
Yes	1.786 (0.943-3.382)	0.075		0.892 (0.497-1.602)	0.703	
Postoperative radiotherapy						
No	1.0 (reference)			1.0 (reference)		
Yes	1.457 (0.726-2.924)	0.290		1.713 (0.903-3.251)	0.100	
Postoperative chemotherapy						
No	1.0 (reference)			1.0 (reference)		
Yes	0.462 (0.221-0.965)	0.040*		1.323 (0.684-2.560)	0.405	
CD4 in TLS						
low	1.0 (reference)			1.0 (reference)		
high	0.317 (0.135-0.746)	0.008*		0.484 (0.213-1.099)	0.083	
CD4+CD8 in TLS						
low	1.0 (reference)			1.0 (reference)		
high	0.517 (0.235-1.141)	0.102		0.624 (0.314-1.237)	0.177	
Treg in TLS						
low	1.0 (reference)			1.0 (reference)		
high	1.215 (0.529-2.791)	0.645		0.787 (0.370-1.673)	0.533	

*Wald p Value.

## Discussion

The 5-year survival rate of esophageal cancer reported in epidemiological investigation is only about 30% (33, 34). In recent years, with increased health education on cancer prevention, improved treatment and early diagnosis, the survival rate of cancer patients has been improving year by year. In this study, the 5-year survival rate of patients after esophagectomy has reached 61%. Although TNM stage is the best reference index for predicting tumor prognosis, it also has its limitations. The 8th edition of AJCC staging was used in this cohort, and N1, N2, N3 staging did not well reflect its predictive value. The lymph node metastasis (N0 or Nx) had an impact on prognosis, but the degree of N metastasis (Nx) had no impact on prognosis, as shown in **Supplementary Figure 1A**. In the 8^th^ edition of AJCC tumor staging, T stage and N stage were adjusted. Some stage III cases with lymph node metastasis in the old edition were reclassified as stage IV, which led to the survival rate of stage IV cases in this study being higher than that reported in the previous literature (35, 36).

Tumor related immune evaluation is a new perspective of tumor prognosis. Yasushi Cho et al. found that the better prognosis of ESCC patients was related to the number of CD4^+^ and CD8^+^ T cells in the matrix and the number of CD8^+^T cells in the cancer cell nest. In addition, the number of CD8 ^+^ T cells in the matrix and the cancer cell nest is related to one another, and the synergy between CD4^+^and CD8^+^ T cells is closely related to the prognosis of ESCC patients (37). Zheng, Y. et al. performed single-cell sequencing and found that exhausted T cells, NK cells, and regulatory T cells (Tregs), alternately activated macrophages and tolerant dendritic cells played a dominant role in TME. They also found that CD8 T cells showed continuous progression from pre-depleted to depleted T cells (38). However, in our study, we concentrated on investigating the status of total T cells and Treg cells, and the dynamic changes of T cells were not specifically reflected in our experiment. Shi K et al. employed single-cell RNA sequencing to evaluateTME, and found that the infiltration of myofibroblasts might be involved in the progress of ESCC. They also found a variety of cell subtypes of T cells and myeloid cells, including tumor-enriched HAVCR2+CD4+T cells with significant depletion. This study provides in-depth insights into the cell heterogeneity of TME in ESCC, suggesting the complexity of multiple cell participation in TME (39). In previous studies, high number of infiltrating CD8 effector T cells were thought to be associated with better prognosis (40–42). In a multicenter study of colorectal cancer, CD3 and CD8 T lymphocytes were statistically analyzed in the tumor center and invasive edge. It was found that the higher the density of CD8 infiltrated at the tumor edge, the better the survival and prognosis of patients (13). However, Chaloner, B. R., et al. detected different T cell subtypes and their relationship with survival times in gastric cancer tissue with multiple fluorescence, and found that CD45RO-cell and FOXP3-cell densities were significantly related to tumor-specific survival. However, CD8 showed no statistical significance, which was similar to our finding (43). In our study, high infiltration density of CD8 in tumor predicts a better survival prognosis but the difference was not statistically significant. However, we could not rule out the possibility that this was due to the number of cases examined in this study.

In the survival analysis and comparison of the data in serum IgG4 and tissue IgG4 positive cells, only serum IgG4 correlated to poor prognosis. The relationship between the number of IgG4 positive cells and prognosis is not definite. Even in cases with lymph node metastasis, the higher the density of IgG4 positive cells, the better the survival and prognosis of patients. The results are shown in the attached **Supplementary Figure 1B**. Specifically, IgG4 is synthesized in B lymphocytes and released into blood or tissue fluid to play its immune function. This is an important feature of B cell-mediated immunity. We observed that IgG4 was mostly located in cell cytoplasm, and in some cases IgG4 positive signal was detected in the serum within blood vessels (**Supplementary Figure 2**). This phenomenon was found in approximately 30% of cases. The concentration of IgG4 in TME seems more important for exertion of actions than numbers of cells that contain IgG4. The number of IgG4 positive cells in TME is not necessarily proportional to the local concentration of IgG4 as it could be produced by B lymphocytes elsewhere. This could explain the fact that serum IgG4 concentration is more closely related to patient survival than the number of IgG4 positive B lymphocytes in TME. However, it should be pointed out that since the serum samples and tissue samples examined in this study were not from the exactly same group of patients, although the patients had considerable overlapping, we could not rule out the possibility that the difference in survival time between the two groups of patients used to measure serum concentration and cell density in tumor tissue was caused by the difference of the two cohort of patients.

The density of IgG4 plasma cells is positively correlated to that of CD4 T cells in this study. IL-10 is an anti-inflammatory factor that can promote the synthesis of IgG4 (44, 45). IL-10 has strong anti-inflammatory function, which is thought to be secreted mainly by Th2 cells. In recent years, it has also been reported that Th1 cells could regulate the expression of IL-10 through Notch pathway (46), suggesting that Th1 and Th2 might have similar characteristics in the secretion of inflammatory cytokines. In this study we found that CD4 produced a large amount of IL-10 in TLS of the tumor which suggests that CD4 may regulate B cell IgG4 production by up regulating the level of IL-10 (**Supplementary Figure 3**). The differentiation between Th1 and Th2 cells is usually based on IL-4 and IFN- γ secretion (47). Our results are not sufficient to ascertain that IL-10 positive cells belong to Th1 or Th2 type. Among the inflammatory cells recruited in TME, T cells play an important role in cellular immunity. CD4-positive Th cells secreted a large number of anti-inflammatory factors such as IL-10, which may promote the conversion of B cells to plasma cells with IgG4 expression. It appears that cellular immunity and humoral immunity may exchange information through cytokines.

Ethanol induces chemical burns on the surface of esophageal mucosa and affects the microbial homeostasis of oral cavity and esophagus (48, 49), and also leads to the damage of esophageal mucosal barrier (50). In addition, the cumulative damage of nitrite in pickled fish, the accumulation of tea or noodle soup with high temperature, genetic susceptibility may be the cause of esophageal cancer (3). Interestingly, we found that most of the patients were male and had a long history of tobacco and alcohol intake. Tobacco may be a cause of esophageal cancer, and about 71.2% of ESCC patients of this cohort had history of tobacco use. This phenomenon was also observed in similar statistics of esophageal cancer research (50, 51). However, the history of alcohol intake is a risk factor for the poor prognosis of ESCC in this study, it might not be a critical factor.

## Conclusion

In this study, we examined the prognostic value of five immune cell variables in the tumor microenvironment and classic pathological staging of surgically resected esophageal cancer. The TNM staging appears to be the most reliable parameter for prediction of prognosis. Statistics showed that the newly discovered immune suppressive IgG4 positive cells in TME is not an independent factor for prognosis. However, serum IgG4 concentration could be a useful indicator in evaluating prognosis for ESCC.

## Data availability statement

The original contributions presented in the study are included in the article/**Supplementary Material**. Further inquiries can be directed to the corresponding author.

## Ethics statement

The studies involving human participants were reviewed and approved by Ethics Committee of Shantou University Medical College (SUMC-2021-09). The patients/participants provided their written informed consent to participate in this study.

## Author contributions

JG, HW, LH contributed to the conception and design of the study. CS, ZiL, HW, CM, YX, ZhL, XM, YG, PL and WZ participated in performing experiment, data collection, data analysis and interpretation. XW and CM helped with sample collection. HW was a major contributor in writing the manuscript, and JG revised the manuscript. All authors contributed to the article and approved the submitted version.

## References

[1] Sung H Ferlay J Siegel RL Laversanne M Soerjomataram I Jemal A. Global cancer statistics 2020: GLOBOCAN estimates of incidence and mortality worldwide for 36 cancers in 185 countries. CA Cancer J Clin (2021) 71(3):209–49. doi: 10.3322/caac.21660 33538338

[2] Lin Y Totsuka Y He Y Kikuchi S Qiao Y Ueda J. Epidemiology of esophageal cancer in Japan and China. J Epidemiol (2013) 23(4):233–42. doi: 10.2188/jea.JE20120162 PMC3709543 23629646

[3] Abnet CC Arnold M Wei WQ. Epidemiology of esophageal squamous cell carcinoma. Gastroenterology (2018) 154(2):360–73. doi: 10.1053/j.gastro.2017.08.023 PMC5836473 28823862

[4] Bray F Ferlay J Soerjomataram I Siegel RL Torre LA Jemal A. Global cancer statistics 2018: GLOBOCAN estimates of incidence and mortality worldwide for 36 cancers in 185 countries. CA Cancer J Clin (2018) 68(6):394–424. doi: 10.3322/caac.21492 30207593

[5] Berry MF. Esophageal cancer: staging system and guidelines for staging and treatment. J Thorac disease (2014) 6 Suppl 3(Suppl 3):S289–S97. doi 10.3978/j.issn.2072-1439.2014.03.11 PMC4037413 24876933

[6] Rice TW Patil DT Blackstone EH. 8th edition AJCC/UICC staging of cancers of the esophagus and esophagogastric junction: Application to clinical practice. Ann Cardiothorac Surg (2017) 6(2):119–30. doi: 10.21037/acs.2017.03.14 PMC5387145 28447000

[7] Rice TW. Esophageal cancer staging. Korean J Thorac Cardiovasc Surg (2015) 48(3):157–63. doi: 10.5090/kjtcs.2015.48.3.157 PMC4463223 26078921

[8] Hanahan D Coussens LM. Accessories to the crime: Functions of cells recruited to the tumor microenvironment. Cancer Cell (2012) 21(3):309–22. doi: 10.1016/j.ccr.2012.02.022 22439926

[9] Anderson NM Simon MC. The tumor microenvironment. Curr Biol CB (2020) 30(16):R921–r5. doi: 10.1016/j.cub.2020.06.081 PMC8194051 32810447

[10] Whiteside TL. The tumor microenvironment and its role in promoting tumor growth. Oncogene (2008) 27(45):5904–12. doi: 10.1038/onc.2008.271 PMC3689267 18836471

[11] Galon J Pagès F Marincola FM Thurin M Trinchieri G Fox BA. The immune score as a new possible approach for the classification of cancer. J Trans Med (2012) 10:1. doi: 10.1186/1479-5876-10-1 PMC3269368 22214470

[12] Galon J Hermitte F Mlecnik B Marliot F Bifulco CB Lugli A. Immunoscore clinical utility to identify good prognostic colon cancer stage II patients with high-risk clinico-pathological features for whom adjuvant treatment may be avoided. J Clin Oncol (2019) 37(4_suppl):487. doi: 10.1200/JCO.2019.37.4_suppl.487

[13] Pages F Mlecnik B Marliot F Bindea G Ou FS Bifulco C. International validation of the consensus immunoscore for the classification of colon cancer: A prognostic and accuracy study. Lancet (2018) 391(10135):2128–39. doi: 10.1016/S0140-6736(18)30789-X 29754777

[14] Angell HK Bruni D Barrett JC Herbst R Galon J. The immunoscore: Colon cancer and beyond. Clin Cancer Res (2020) 26(2):332. doi: 10.1158/1078-0432.CCR-18-1851 31413009

[15] Chen L Han X. Anti–PD-1/PD-L1 therapy of human cancer: Past, present, and future. J 321 Clin Invest (2015) 125(9):3384–91. doi: 10.1172/JCI80011 PMC4588282 26325035

[16] Liu J Chen Z Li Y Zhao W Wu J Zhang Z. PD-1/PD-L1 checkpoint inhibitors in tumor immunotherapy. Front Pharmacol (2021) 12:731798. doi: 10.3389/fphar.2021.731798 34539412 PMC8440961

[17] Rowshanravan B Halliday N Sansom DM. CTLA-4: A moving target in immunotherapy. Blood (2018) 131(1):58–67. doi: 10.1182/blood-2017-06-741033 29118008 PMC6317697

[18] Lei X Lei Y Li JK Du WX Li RG Yang J. Immune cells within the tumor microenvironment: Biological functions and roles in cancer immunotherapy. Cancer letters (2020) 470:126–33. doi: 10.1016/j.canlet.2019.11.009 31730903

[19] Bruni D Angell HK Galon J. The immune contexture and immunoscore in cancer prognosis and therapeutic efficacy. Nat Rev Cancer (2020) 20(11):662–80. doi: 10.1038/s41568-020-0285-7 32753728

[20] Agresti A Vercelli D. Analysis of gamma4 germline transcription in human b cells. Int Arch Allergy Immunol (1999) 118(2-4):279–81. doi: 10.1159/000024099 10224410

[21] van der Neut Kolfschoten M Schuurman J Losen M Bleeker WK Martínez-Martínez P Vermeulen E. Anti-inflammatory activity of human IgG4 antibodies by dynamic fab arm exchange. Science (2007) 317(5844):1554. doi: 10.1126/science.1144603 17872445

[22] Aalberse RC Schuurman J. IgG4 breaking the rules. Immunology (2002) 105(1):9–19. doi: 10.1046/j.0019-2805.2001.01341.x 11849310 PMC1782638

[23] Crescioli S Correa I Karagiannis P Davies AM Sutton BJ Nestle FO. IgG4 characteristics and functions in cancer immunity. Curr Allergy Asthma Rep (2016) 16(1):7. doi: 10.1007/s11882-015-0580-7 26742760 PMC4705142

[24] Miyatani K Saito H Murakami Y Watanabe J Kuroda H Matsunaga T. A high number of IgG4-positive cells in gastric cancer tissue is associated with tumor progression and poor prognosis. Virchows Archiv an Int J pathology (2016) 468(5):549–57. doi: 10.1007/s00428-016-1914-0 26951261

[25] Karagiannis P Villanova F Josephs DH Correa I Van Hemelrijck M Hobbs C. Elevated IgG4 in patient circulation is associated with the risk of disease progression in melanoma. Oncoimmunology (2015) 4(11):e1032492. doi: 10.1080/2162402X.2015.1032492 26451312 PMC4590000

[26] Wang H Xu Q Zhao C Zhu Z Zhu X Zhou J. An immune evasion mechanism with IgG4 playing an essential role in cancer and implication for immunotherapy. J immunotherapy Cancer (2020) 8(2):e000661 doi: 10.1136/jitc-2020-000661 PMC7443307 32819973

[27] Harada K Shimoda S Kimura Y Sato Y Ikeda H Igarashi S. Significance of immunoglobulin G4 (IgG4)-positive cells in extrahepatic cholangiocarcinoma: Molecular mechanism of IgG4 reaction in cancer tissue. Hepatol (Baltimore Md) (2012) 56(1):157–64. doi: 10.1002/hep.25627 22290731

[28] Nakashima H Miyake K Moriyama M Tanaka A Watanabe M Abe Y. An amplification of IL-10 and TGF-beta in patients with IgG4-related tubulointerstitial nephritis. Clin nephrology (2010) 73(5):385–91. doi: 10.5414/CNP73385 20420800

[29] Couper KN Blount DG Riley EM. IL-10: the master regulator of immunity to infection. J Immunol (Baltimore Md 1950) (2008) 180(9):5771–7. doi: 10.4049/jimmunol.180.9.5771 18424693

[30] Wang H Zhou J Li J Geng Y Meng P Ma C. A study of multinucleated giant cells in esophageal cancer. Clin Immunol (2021) 222:108600. doi: 10.1016/j.clim.2020.108600 33197619

[31] St. Paul M Ohashi PS. The roles of CD8+ T cell subsets in antitumor immunity. Trends Cell Biol (2020) 30(9):695–704. doi: 10.1016/j.tcb.2020.06.003 32624246

[32] Sakaguchi S Yamaguchi T Nomura T Ono M. Regulatory T cells and immune tolerance. Cell (2008) 133(5):775–87. doi: 10.1016/j.cell.2008.05.009 18510923

[33] Shen J Kong M Yang H Jin K Chen Y Fang W. Pathological complete response after neoadjuvant treatment determines survival in esophageal squamous cell carcinoma patients (NEOCRTEC5010). Ann Trans Med (2021) 9(20):1516. doi: 10.21037/atm-21-3331 PMC8576689 34790722

[34] Arnold M Abnet CC Neale RE Vignat J Giovannucci EL McGlynn KA. Global burden of 5 major types of gastrointestinal cancer. Gastroenterology (2020) 159(1):335–49.e15. doi: 10.1053/j.gastro.2020.02.068 32247694 PMC8630546

[35] Zhang D Zheng Y Wang Z Huang Q Cao X Wang F. Comparison of the 7th and proposed 8th editions of the AJCC/UICC TNM staging system for esophageal squamous cell carcinoma underwent radical surgery. Eur J Surg Oncol (2017) 43(10):1949–55. doi: 10.1016/j.ejso.2017.06.005 28716377

[36] Park SY Kim DJ Suh JW Byun GE. Comparison of the 11(th) Japanese classification and the AJCC 7(th) and 8(th) staging systems in esophageal squamous cell carcinoma patients. J Thorac disease (2018) 10(8):5039–46. doi: 10.21037/jtd.2018.07.48 PMC6129869 30233878

[37] Cho Y Miyamoto M Kato K Fukunaga A Shichinohe T Kawarada Y. CD4+ and CD8+ T cells cooperate to improve prognosis of patients with esophageal squamous cell carcinoma. Cancer Res (2003) 63(7):1555–9. 12670904

[38] Zheng Y Chen Z Han Y Han L Zou X Zhou B. Immune suppressive landscape in the human esophageal squamous cell carcinoma microenvironment. Nat Commun (2020) 11(1):6268. doi: 10.1038/s41467-020-20019-0 33293583 PMC7722722

[39] Shi K Li Y Yang L Zhang Z Guo D Zhang J. Profiling transcriptional heterogeneity of epithelium, fibroblasts, and immune cells in esophageal squamous cell carcinoma by single-cell RNA sequencing. FASEB J (2022) 36(11):e22620. doi: 10.1096/fj.202200898R 36260317

[40] Ma L Sun L Zhao K Dong Z Huang Z Meng X. The prognostic value of TCF1+CD8+T in primary small cell carcinoma of the esophagus. Cancer Sci (2021) 112(12):4968–76. doi: 10.1111/cas.15167 PMC8645743 34657342

[41] Goode EL Block MS Kalli KR Vierkant RA Chen W Fogarty ZC. Dose-response association of CD8+ tumor-infiltrating lymphocytes and survival time in high-grade serous ovarian cancer. JAMA Oncol (2017) 3(12):e173290. doi 10.1001/jamaoncol.2017.3290 29049607 PMC5744673

[42] Sato E Olson SH Ahn J Bundy B Nishikawa H Qian F. Intraepithelial CD8+ tumor-infiltrating lymphocytes and a high CD8+/regulatory T cell ratio are associated with favorable prognosis in ovarian cancer. Proc Natl Acad Sci U S A (2005) 102(51):18538–43. doi: 10.1073/pnas.0509182102 PMC1311741 16344461

[43] Challoner BR von Loga K Woolston A Griffiths B Sivamanoharan N Semiannikova M. Computational image analysis of T-cell infiltrates in resectable gastric cancer: Association with survival and molecular subtypes. J Natl Cancer Institute (2021) 113(1):88–98. doi: 10.1093/jnci/djaa051 PMC7781469 32324860

[44] van de Veen W Stanic B Yaman G Wawrzyniak M Söllner S Akdis DG. IgG4 production is confined to human IL-10-producing regulatory b cells that suppress antigen-specific immune responses. J Allergy Clin Immunol (2013) 131(4):1204–12. doi: 10.1016/j.jaci.2013.01.014 23453135

[45] Jeannin P Lecoanet S Delneste Y Gauchat JF Bonnefoy JY. IgE versus IgG4 production can be differentially regulated by IL-10. J Immunol (Baltimore Md 1950) (1998) 160(7):3555–61. doi: 10.4049/jimmunol.160.7.3555 9531318

[46] Rutz S Janke M Kassner N Hohnstein T Krueger M Scheffold A. Notch regulates IL-10 production by T helper 1 cells. Proc Natl Acad Sci U S A (2008) 105(9):3497–502. doi: 10.1073/pnas.0712102105 PMC2265185 18292228

[47] Zhou L Chong MM Littman DR. Plasticity of CD4+ T cell lineage differentiation. Immunity (2009) 30(5):646–55. doi: 10.1016/j.immuni.2009.05.001 19464987

[48] Abreu MT Peek RM Jr. Gastrointestinal malignancy and the microbiome. Gastroenterology (2014) 146(6):1534–46.e3. doi: 10.1053/j.gastro.2014.01.001 24406471 PMC3995897

[49] Kawasaki M Ikeda Y Ikeda E Takahashi M Tanaka D Nakajima Y. Oral infectious bacteria in dental plaque and saliva as risk factors in patients with esophageal cancer. Cancer (2021) 127(4):512–9. doi: 10.1002/cncr.33316 33156979

[50] Dong J Thrift AP. Alcohol, smoking and risk of oesophago-gastric cancer. Best Pract Res Clin gastroenterology (2017) 31(5):509–17. doi: 10.1016/j.bpg.2017.09.002 29195670

[51] Li J Xu J Zheng Y Gao Y He S Li H. Esophageal cancer: Epidemiology, risk factors and screening. Chin J Cancer Res (2021) 33(5):535–47. doi: 10.21147/j.issn.1000-9604.2021.05.01 PMC8580797 34815628

