# Supplementary material for: Evaluation of multiple immune cells and patient outcomes in esophageal squamous cell carcinoma
Source: Front Immunol. 2023 Feb 20;14:1091098. doi: 10.3389/fimmu.2023.1091098 (PMC9986480; doi:10.3389/fimmu.2023.1091098)
Supplement: Supplementary file 1 [file DataSheet_1.docx]

Supplementary Material

**Supplementary Table 1. Antibody information**


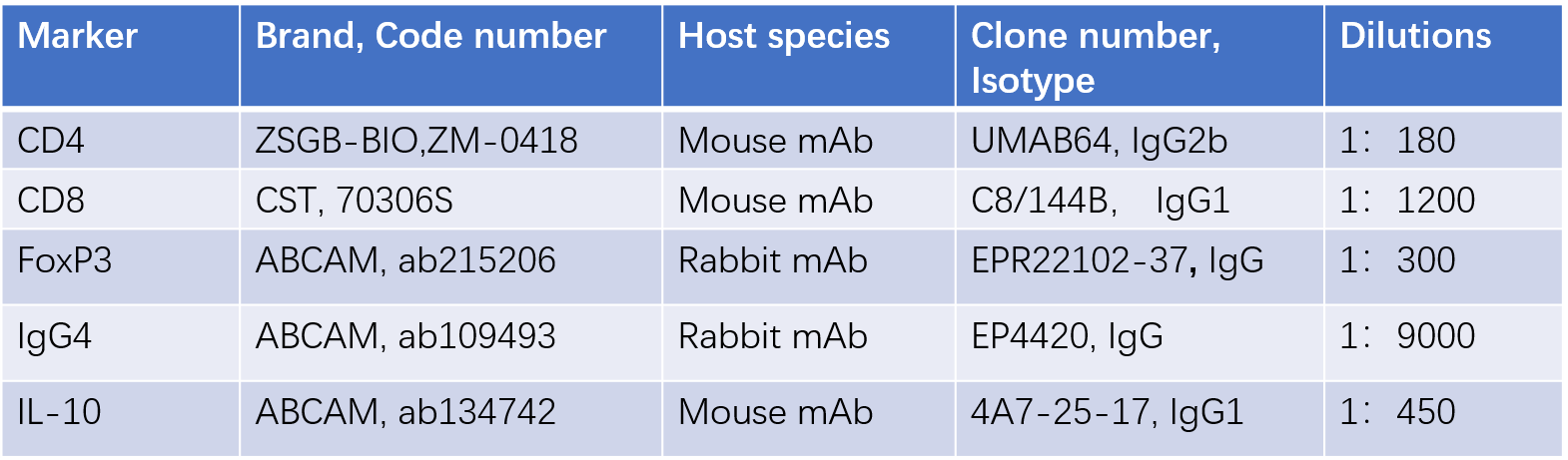


**Supplementary Table 2. Mean/ median survival time**

**
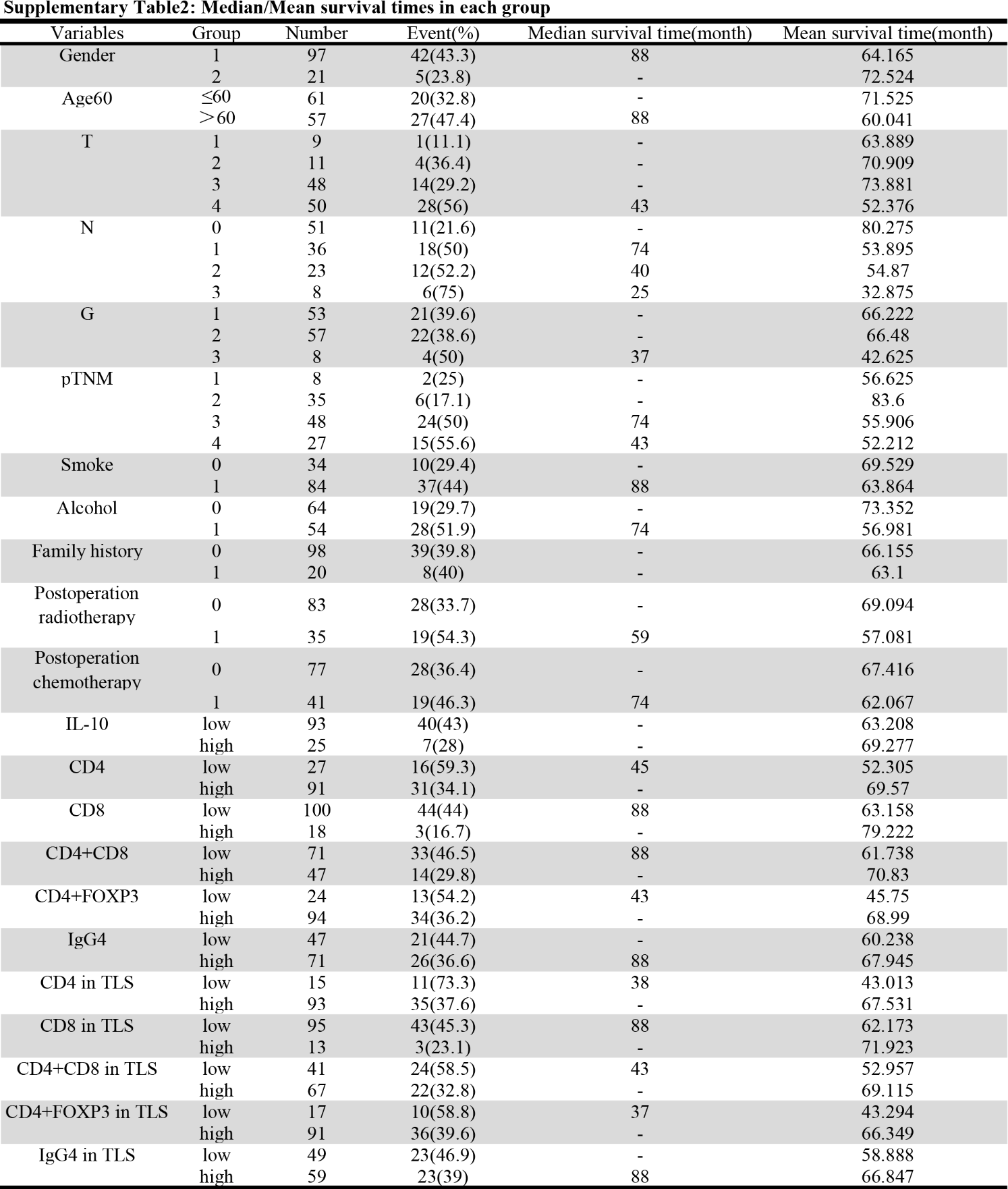
**


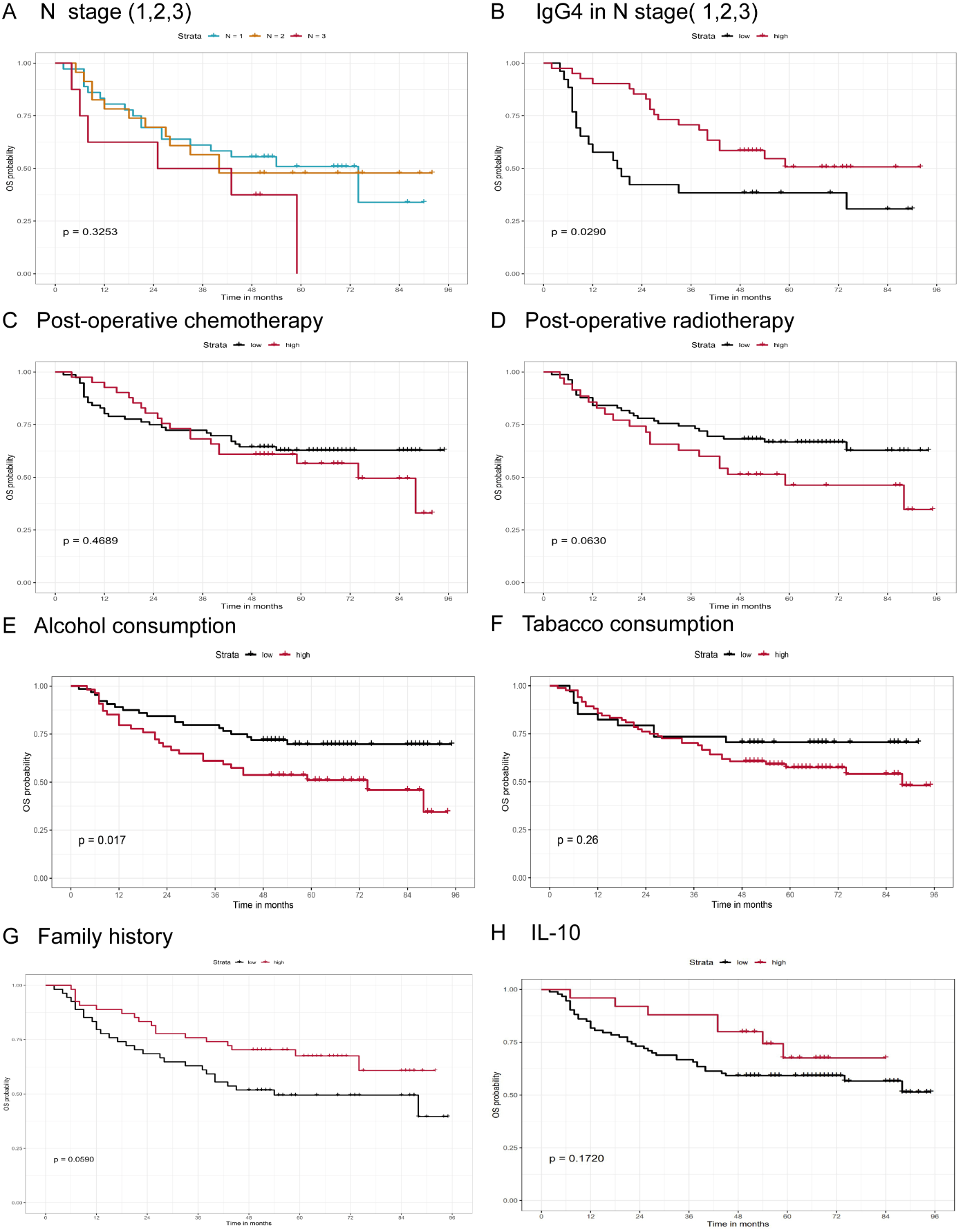


**Supplementary Figure 1.** Kaplan-Meire survival analysis. **A** Comparing survival of 118 ESCC patients in 3 different lymph node metastasis stage (p=0.32, ns). **B** The survival time of high IgG4^+^ cells infiltration (>10 cells) was better in those patients with lymph node metastasis (p=0.02, *). **C, D** Both postoperative chemotherapy or radiotherapy had no statistical significance in ESCC patients’ survival analysis. **E, F** Alcohol consumption was related to shorter survival time and poor prognosis (p=0.01, *). While tobacco use could be a cause for developing ESCC (71.2%), it does not seem to be a significant predictive factor for survival once diagnosed (p=0.26, ns). G, H Analysis of family history (p=0.059, ns) and IL-10 expression in tumor microenvironment (p=0.17, ns).

**
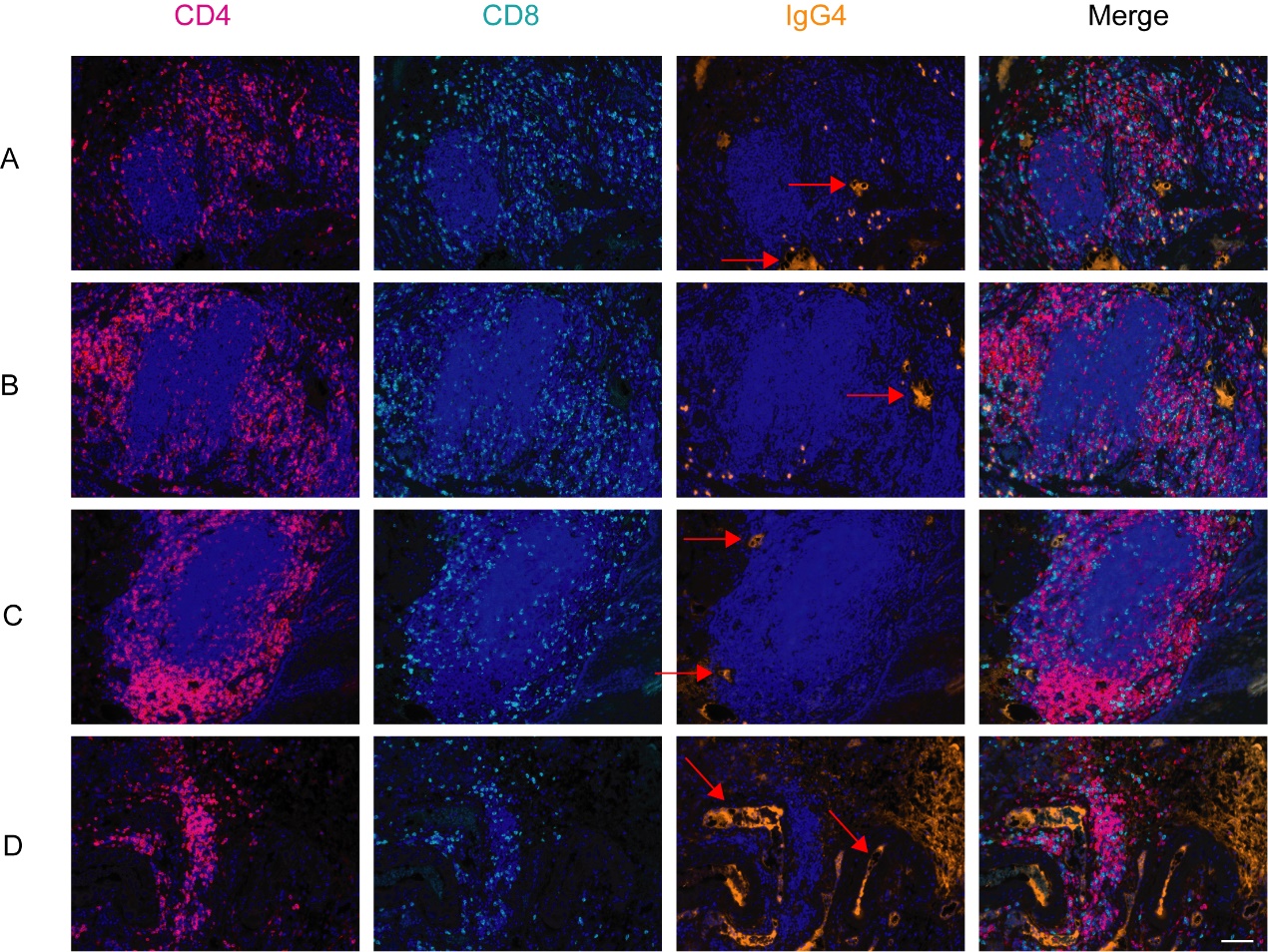
**

**Supplementary Figure 2.** Typical image of positive IgG4 staining in vascular space (red arrow). **A, B, C, D** each shows CD4 (rose red), CD8 (light blue), IgG4 (orange) positive staining and an integrated image. Scale bar=60μm.

**
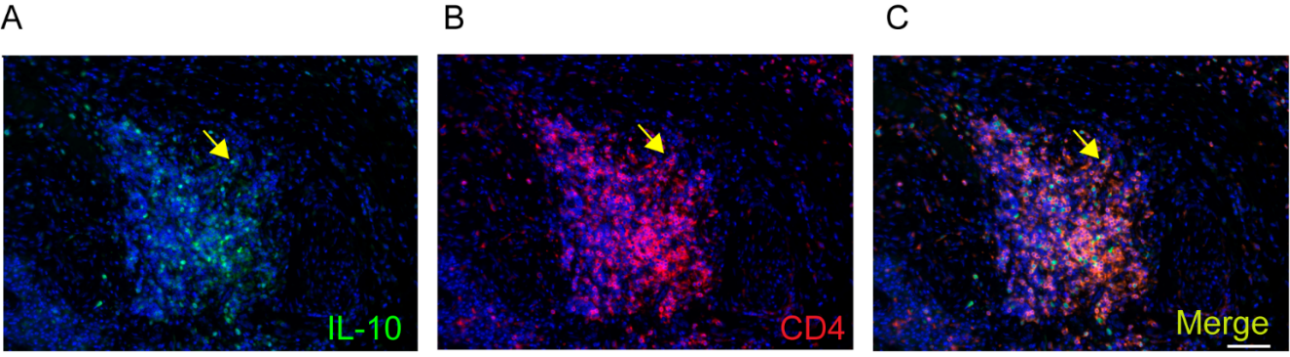
**

**Supplementary Figure3.**  **A, B, C** CD4^+^ T cells overlay with IL-10 cells, suggesting part of CD4^+^ T cells expressing IL-10. Scale bar=60μm.
